# Outcome prediction by interim positron emission tomography and IgM monoclonal gammopathy in diffuse large B-cell lymphoma

**DOI:** 10.1007/s00277-023-05393-1

**Published:** 2023-08-11

**Authors:** Patricia Johansson, Stefan Alig, Julia Richter, Christine Hanoun, Jan Rekowski, Jan Dürig, Bauke Ylstra, Daphne de Jong, Wolfram Klapper, Ash A. Alizadeh, Ulrich Dührsen, Andreas Hüttmann

**Affiliations:** 1 Department of Hematology, West German Cancer Center, University Hospital Essen, University of Duisburg-Essen, Hufelandstraße 55, 45147 Essen, Germany; 2 Institute of Cell Biology (Cancer Research), Faculty of Medicine, University of Duisburg-Essen, Essen, Germany; 3 Department of Medicine, Divisions of Oncology and Hematology, Stanford University, Stanford, CA USA; 4 Department of Hematopathology, University Hospital Schleswig-Holstein, Kiel, Germany; 5 Institute for Medical Informatics, Biometry and Epidemiology, University Hospital Essen, University of Duisburg-Essen, Essen, Germany; 6 Department of Pathology, Cancer Center Amsterdam, Amsterdam UMC, Vrije Universiteit Amsterdam, Amsterdam, The Netherlands; 7 Stanford Cancer Institute, Institute for Stem Cell Biology & Regenerative Medicine, Stanford, CA USA

**Keywords:** Diffuse large B-cell lymphoma, Positron emission tomography, IgM gammopathy, Outcome prediction, Survival

## Abstract

In diffuse large B-cell lymphoma (DLBCL), a positive interim positron emission tomography (PET) scan predicts treatment failure, but the proportion of high-risk patients thus identified is small. To improve prediction, we combined the interim PET result with the presence or absence of an associated IgM gammopathy. Of 108 DLBCL patients participating in a prospective trial, nine (8%) were interim PET positive and 19 (18%) had an IgM gammopathy. The monoclonal protein was not associated with distinguishing genetic features, and its light chain restriction was not always concordant with the light chain restriction of the lymphoma. The information provided by interim PET and IgM gammopathy was combined to dichotomize the population into sizeable high-risk (1–2 adverse factors) and low-risk groups (no adverse factor) with widely different outcomes (population size, 25% vs. 75%; 3-year risk of progression, 51% vs. 10%; 3-year overall survival, 64% vs. 95%). Multivariable analyses including established risk factors revealed the interim PET result and the IgM gammopathy status to be the only factors significantly associated with outcome. Information about interim PET response and IgM gammopathy may be useful in studies testing risk-adapted treatment strategies.

**Supplementary Information:**

The online version contains supplementary material available at 10.1007/s00277-023-05393-1.

## Introduction

Diffuse large B-cell lymphoma (DLBCL) is the most common cancer of the immune system. It can be cured by immunochemotherapy in about two-thirds of cases [1]. Prognostication for individual patients is based on clinical features and molecular markers. The most commonly used tool is the International Prognostic Index (IPI) which is based on five clinical factors subdividing the DLBCL population into four prognostic groups with 3-year survival rates between 60 and 90% [2].

18[F] Fluorodeoxyglucose (FDG) positron emission tomography/computed tomography (PET/CT) is the imaging modality of choice to define pretreatment lymphoma dissemination and post-treatment remission status. It can also be used to predict outcome. Baseline total metabolic tumor volume (TMTV), largely representing tumor mass, is of paramount importance, outperforming the IPI [3, 4]. Early response assessment by interim PET, representing chemotherapy sensitivity, provides prognostic information independent of baseline factors [5]. This was confirmed in the “Positron Emission Tomography–Guided Therapy of Aggressive Non-Hodgkin Lymphomas” (PETAL) trial that set out—and failed—to improve outcome by switching patients with an insufficient response to the first two cycles of rituximab, cyclophosphamide, doxorubicin, vincristine, and prednisone (R-CHOP) to a more intense therapy [6].

Similar to other types of B-cell lymphoma, DLBCL can be associated with a monoclonal immunoglobulin. The most common type in Western Europe, IgM gammopathy, has been associated with poor outcome [7–10]. It is detected with comparable sensitivity by immunofixation or the Hevylite^TM^ assay that allows distinction between *μ* heavy chains linked to *κ* as opposed to *λ* light chains [11]. Similar to the Freelite^TM^ assay measuring unbound *κ* and *λ* light chains [12, 13], calculated ratios between IgM-*κ* and IgM-*λ* outside the reference range indicate a monoclonal gammopathy [7].

Interim PET is a suitable method to identify chemotherapy-refractory patients [6], but the proportion of patients thus detected is small and the majority of DLBCL patients eventually failing therapy have a favorable interim PET scan [14]. To improve outcome prediction, we assessed the prognostic impact of free light chain and IgM abnormalities on DLBCL outcome in a post hoc analysis of the PETAL trial and correlated the findings with FDG-PET/CT data and molecular markers detected in tumor biopsies and circulating tumor DNA (ctDNA).

## Materials and methods

### Study design

The PETAL trial (ClinicalTrials.gov NCT00554164; EudraCT 2006-001641-33) was a multicenter study for newly diagnosed aggressive non-Hodgkin lymphomas [6]. The study was approved by the Federal Institute for Drugs and Medical Devices and the ethics committees of the participating sites. All patients gave written informed consent including permission of data use for post hoc scientific analyses.

In the first two cycles, the patients were uniformly treated with R-CHOP. Interim PET was performed a median of 20 days after cycle 2. Patients with a favorable response (“negative” PET scan) received four more cycles of R-CHOP or the same treatment plus two extra doses of rituximab. Patients with an unfavorable response (“positive” PET scan) were randomly assigned to receive six additional cycles of R-CHOP or six blocks of a more intense, methotrexate- and hyperfractionated alkylator–based protocol originally developed for the treatment of Burkitt’s lymphoma [15]. Radiotherapy was not included in the study protocol. Since interim PET-related treatment changes failed to have an impact on outcome [6, 14], all study arms were combined in the present analysis.

Prospective collection of baseline blood samples for scientific analyses was restricted to patients recruited in the final two years of the trial.

### FDG-PET/CT imaging and evaluation

The imaging conditions have been described previously [6]. The treatment response was determined by dividing the maximum standardized uptake value (SUV_max_) of the hottest residual lesion on the interim scan by the SUV_max_ of the hottest lesion on the baseline scan (ΔSUV_max_). Negative scans were defined by complete disappearance of all non-physiological FDG activity or SUV_max_ reduction by >66% [16]. In the PETAL trial, the ΔSUV_max_ procedure proved highly reproducible [6] and superior to the Deauville 5-point scale for outcome prediction [16] which was subsequently confirmed in a larger study [17]. Compared to the ΔSUV_max_ procedure with a cut-off value of 66%, considering scores 4 and 5 of the Deauville scale as high risk yields a higher proportion of false positive results and considering only score 5 as high risk yields a lower number of high-risk patients [16, 17]. TMTV was determined centrally on archived PET/CT scans, using the 41% SUV_max_ method [3]. Because end-of-treatment PET scans were not financed in the trial, end-of-treatment responses were defined by CT criteria [6, 18].

### Freelite ^TM^ and Hevylite^TM^ assays

The Freelite^TM^ and Hevylite^TM^ assays (The Binding Site Ltd., Birmingham, UK) were performed at the Central Laboratory of the University Hospital of Essen to measure serum free *κ*, free *λ*, IgM-*κ*, and IgM-*λ* concentrations and calculate the respective ratios [7, 11–13]. Normal concentrations and ratios were defined according to the manufacturer’s recommendations (*κ*, 3.3–19.4 mg/L; *λ*, 5.7–26.3 mg/L; *κ*:*λ* ratio, 0.26–1.65; IgM-*κ*, 0.19–1.63 g/L; IgM-*κ*, 0.12–1.01 g/L; IgM-*κ*:IgM-*λ* ratio, 1.18–2.74).

### Tumor biopsies

To group the lymphomas according to the cell-of-origin (COO) classification [19], available formalin-fixed paraffin-embedded (FFPE) tumor biopsies were analyzed by mRNA-based gene expression using the HTG EdgeSeq System (HTG Molecular Diagnostics, Tucson, AZ, USA) and by immunohistochemistry using the Hans classifier [20]. For IgM and light chain expression, full tissue slides were stained with suitable antibodies (IgM, clone 0425, dilution 1:5000; *κ*, clone A0191, dilution 1:15,000; *λ*, clone A0193, dilution 1:14,000; all from DAKO, Glostrup, Denmark) and evaluated visually by an experienced hematopathologist (WK) for specific cytoplasmic and/or membranous staining, with tonsil tissue serving as a positive control. *MYC*, *BCL2*, and *BCL6* translocations were assessed by fluorescence in situ hybridization (FISH; Vysis-Abbott, Des Plaines, IL, USA) [21]. Chromosomal copy number aberrations were identified by shallow whole genome sequencing [22], and mutations by whole exome sequencing of DNA extracted from available FFPE tumor biopsies (manuscript submitted).

### Circulating tumor DNA

ctDNA was isolated from pretreatment plasma samples using Qiagen’s QIAamp Circulating Nucleic Acid Kit (Qiagen, Germantown, MD, USA) and evaluated by CAPP-Seq as previously described [23–26]. Quantitative levels of ctDNA were measured in haploid genome equivalents per milliliter (hGE/mL), determined as the product of total cell-free DNA concentration and the mean allele fraction of somatic mutations, expressed in log scale (log hGE/mL) [25].

### Mutation calling and LymphGen classification

LymphGen subtypes [27] were inferred using the LymphGen online tool (https://llmpp.nih.gov/lymphgen/index.php) from targeted sequencing of either tumor or plasma specimens. CAPP-Seq-derived mutation calls were generated using a 608 kb targeted sequencing panel suitable for genetic subclassification [28, 29]. Genome-wide copy number alterations and fusions were called as previously described [30, 31].

### Statistical analysis

All analyses were exploratory, applying a two-sided alpha of 0.05. Frequencies were compared using the chi^2^ test or—in case of low numbers—Fisher’s exact test, and continuous variables were compared using the Mann-Whitney *U* test. Time-to-event end-points were analyzed using the Kaplan-Meier estimator, the log-rank test, and, when adjusting for covariates, Cox proportional hazards regression [32]. All analyses were carried out using IBM SPSS Statistics, version 28.0, Armonk, NY, USA. 

## Results

### Patient, lymphoma, and treatment characteristics

Of 609 DLBCL patients participating in the PETAL trial, 108 had pretreatment blood samples available for immunoglobulin analyses. Patient, lymphoma, and treatment characteristics were typical of DLBCL (Tables 1, 2, and 3, right columns). The majority of patients received 6 cycles of R-CHOP with two extra doses of rituximab. As per discretion of the investigator, four patients received consolidating radiotherapy. The median follow-up was 40.7 months.

**Table 1:** Patient characteristics

	No IgM gammopathy^a^	IgM gammopathy^a^	*p*^b^	Total^a^
Number	89	19		108
Age (years), median (range)	56 (25–79)	63 (42–79)	0.065^c^	58 (25–79)
Male patients	48 (54%)	9 (47%)	0.623	57 (53%)
Clinical risk factors				
Age > 60 years	42 (47%)	11 (58%)	0.455	53 (49%)
ECOG performance status ≥ 2	9 (10%)	5 (26%)	0.069	14 (13%)
Ann Arbor stage III or IV	45 (51%)	14 (74%)	0.079	59 (55%)
≥ 2 extranodal manifestations	22 (25%)	9 (47%)	0.056	31 (29%)
Serum lactate dehydrogenase > upper limit of normal	44 (49%)	14 (74%)	0.076	58 (54%)
B symptoms	23 (26%)	8 (42%)	0.171	31 (29%)
International Prognostic Index				
Low risk	44 (49%)	3 (16%)	0.025^d^	47 (43%)
Low-intermediate risk	16 (18%)	3 (16%)		19 (18%)
High-intermediate risk	17 (19%)	8 (42%)		25 (23%)
High risk	12 (14%)	5 (26%)		17 (16%)
Serum abnormalities				
Elevated immunoglobulin free light chain concentration	29 (33%)	10 (53%)	0.118	39 (36%)
Monoclonal immunoglobulin free light chain	19 (21%)	4 (21%)	1.000	23 (21%)

*ECOG*, Eastern Cooperative Oncology Group

^a^Number of patients affected (% of total number of patients in the respective columns) (unless stated otherwise)

^b^Fisher’s exact test (unless stated otherwise)

^c^Mann-Whitney *U* test

^d^4 × 2 chi^2^ test

**Table 2:** Lymphoma characteristics

	No IgM gammopathy^a^	IgM gammopathy^a^	*p*^b^	Total^a^
Histomorphology				
Diffuse large B-cell lymphoma, not otherwise specified	89	19		108
Centroblastic variant	41 (46%)	6 (32%)	0.700^c^	47 (43%)
Immunoblastic variant	3 (3%)	1 (5%)		4 (4%)
Anaplastic variant	3 (3%)	1 (5%)		4 (4%)
Variant not specified	42 (48%)	11 (58%)		53 (49%)
Gene expression (cell-of-origin classification)				
Non-germinal center B-cell-like (protein expression, Hans classifier)	27/49 (55%)	6/11 (55%)	1.000	33/60 (55%)
Activated B-cell-like (mRNA expression, HTG EdgeSeq Assay)	21/37 (57%)	4/7 (57%)	1.000	25/44 (57%)
Translocations				
MYC	6/50 (12%)	1/10 (10%)	1.000	7/60 (12%)
MYC and BCL2 and/or BCL6 (“double hit”)	4/50 (8%)	1/10 (10%)	1.000	5/60 (8%)
Tumor burden				
Total metabolic tumor volume above prognostic threshold (328 cm^3^)^d^	25/77 (32%)	9/17 (53%)	0.162	34/94 (36%)
Total metabolic tumor volume, cm^3^, median (range)	89 (3–3968)	411 (9–2693)	0.124^e^	123 (3–3968)

*SUV*, standardized uptake value

^a^Number of patients affected/total number of patients analyzed (%) (unless stated otherwise)

^b^Fisher’s exact test (unless stated otherwise)

^c^4 × 2 chi^2^ test

^d^This threshold was associated with the largest time-to-progression difference in a subset of 510 DLBCL patients from the PETAL trial [3]

^e^Mann-Whitney *U* test

**Table 3:** Treatment characteristics

	No IgM gammopathy^a^	IgM gammopathy^a^	*p*	Total^a^
Number	89	19		108
Response after 2 cycles R-CHOP				
Interim PET positive (poor treatment response)	8 (9%)	1 (5%)	1.000^b^	9 (8%)
Treatment allocation				
6 × R-CHOP	12 (13%)^c^	3 (16%)	0.970^d^	15 (14%)
6 × R-CHOP + 2 × R	69 (78%)^c^	15 (79%)		84 (78%)
8 × R-CHOP	3 (3%)	0 (0%)		3 (3%)
2 × R-CHOP + 6 × Burkitt’s lymphoma protocol	5 (6%)^e^	1(5%)		6 (5%)
End-of-treatment remission status				
Complete remission	56 (63%)	13 (68%)	0.019^f^	69 (64%)
Partial remission	25 (28%)	2 (11%)		27 (25%)
Stable disease	0 (0%)	1 (5%)		1 (1%)
Progressive disease	1 (1%)	2 (11%)		3 (3%)
Premature treatment termination	7 (8%)	1 (5%)		8 (7%)
Long-term outcome^g^				
Freedom from progression at 3 years	85.9% ± 3.8%	52.6% ± 11.5%	<0.001	80.0% ± 3.9%
Interim PET negative	89.8% ± 3.4%	55.6% ± 11.7%	<0.001	83.5% ± 3.8%
Interim PET positive	41.7% ± 20.5%	0.0%	0.548	35.6% ± 18.6%
Overall survival at 3 years	93.0% ± 2.8%	62.7% ± 11.2%	<0.001	87.5% ± 3.3%
Interim PET negative	94.9% ± 2.5%	66.2 ± 11.3%	<0.001	89.5% ± 3.1%
Interim PET positive	70.0% ± 18.2%	0.0%	0.113	61.0% ± 18.1%

*CHOP*, cyclophosphamide, doxorubicin, vincristine, prednisone; *PET*, positron emission tomography; *R*, rituximab

^a^Number of patients affected (% of total number of patients in the respective columns) (unless stated otherwise)

^b^Fisher’s exact test

^c^One patient received consolidating radiotherapy

^d^4 × 2 chi^2^ test

^e^Two patients received consolidating radiotherapy

^f^5 × 2 chi^2^ test

^g^Kaplan-Meier estimates, *p* by log-rank test

At baseline, 39 patients (36%) had elevated concentrations of one or both free immunoglobulin light chains, 23 (21%) had a monoclonal free light chain (all *κ*), and 19 (18%) had an IgM monoclonal protein (11 IgM-*κ*, accompanied by a monoclonal free *κ* light chain in 4 cases; 8 IgM-*λ*) (Table 1; for details, see Supplementary Information, Table S1). All biopsies underwent reference pathological review, with protein- or mRNA-based gene expression analysis in 60 and 44 patients, respectively, and FISH-based translocation studies in 60 patients [21]. Eight biopsies underwent mutation and copy number analysis by genome sequencing. Pretreatment ctDNA was obtained from 98 patients. Baseline PET scans for post hoc TMTV measurements were available from 94 patients (Table 2).

### Association of IgM monoclonal gammopathy with disease features

Since light chains failed to be statistically significantly associated with outcome (see below), we focused our analysis on IgM monoclonal gammopathy. IgM gammopathy tended to be associated with old age and unfavorable risk factors, with a statistically significant increase in the proportion of patients with high-intermediate or high IPI risk (Table 1). A large tumor burden, defined as TMTV >328 cm^3^ [3], and elevated free light chain concentrations tended to be more frequent in patients with than in patients without IgM gammopathy (Tables 1 and 2).

**Fig. 1:**
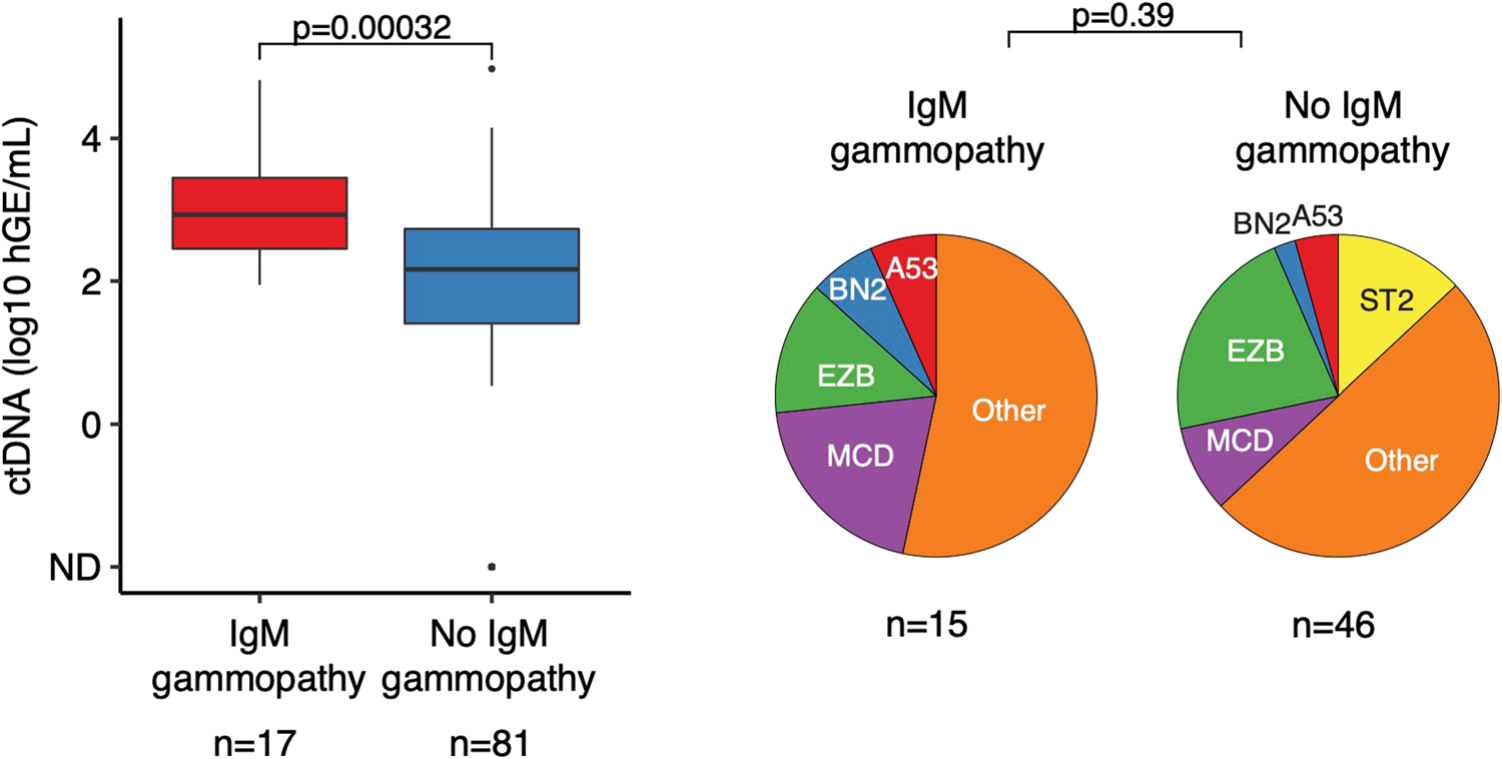
Levels of circulating tumor DNA (ctDNA) in diffuse large B-cell lymphoma patients with or without IgM monoclonal gammopathy (left) and assignment of the lymphomas to the mutation groups defined by the LymphGen classification [27] (right). hGE/mL, haploid genome equivalents per milliliter; ND, not detected

There were no statistically significant differences in histomorphology, COO subtype, or *MYC* translocations between IgM gammopathy–positive and IgM gammopathy–negative lymphomas (Table 2). ctDNA levels were significantly higher in patients with than in patients without IgM gammopathy (Fig. 1, left). Comprehensive mutation analyses were performed in 61 patients with available tumor sequencing (*n*=8) or plasma specimens with sufficiently high ctDNA levels (*n*=53). In univariable analysis, no individual gene was significantly differentially mutated between patients with or without IgM gammopathy (for details, see Supplementary Information, Table S2). Of note, mutations in *TP53* or *MYD88* recently reported to occur in 31% and 31% of IgM gammopathy–positive DLBCL [10], were observed in 13% and 20% of lymphomas with IgM gammopathy as compared to 11% and 17% of cases without IgM gammopathy. Mutations in *CD79B* reported to occur in 23% of IgM gammopathy–positive DLBCL [10], were found in 20% of lymphomas with IgM gammopathy and in 13% of lymphomas without IgM gammopathy, with 13% and 7% of the lymphomas harboring both a CD79B and a MYD88 mutation. Based on the identified alterations, about half the cases were successfully classified into non-other subtypes according to the LymphGen classification [27]. There were no statistically significant differences in class assignment between patients with or without IgM gammopathy (Fig. 1, right).

By immunohistochemistry, four of seven available biopsies from patients with IgM gammopathy expressed IgM and three expressed light chains (2 *κ*, 1 *λ*). In only one case was the light chain restriction in the lymphoma concordant with the light chain of the monoclonal serum protein (*κ*).

### Treatment results in relation to interim PET and IgM monoclonal gammopathy

**Fig. 2:**
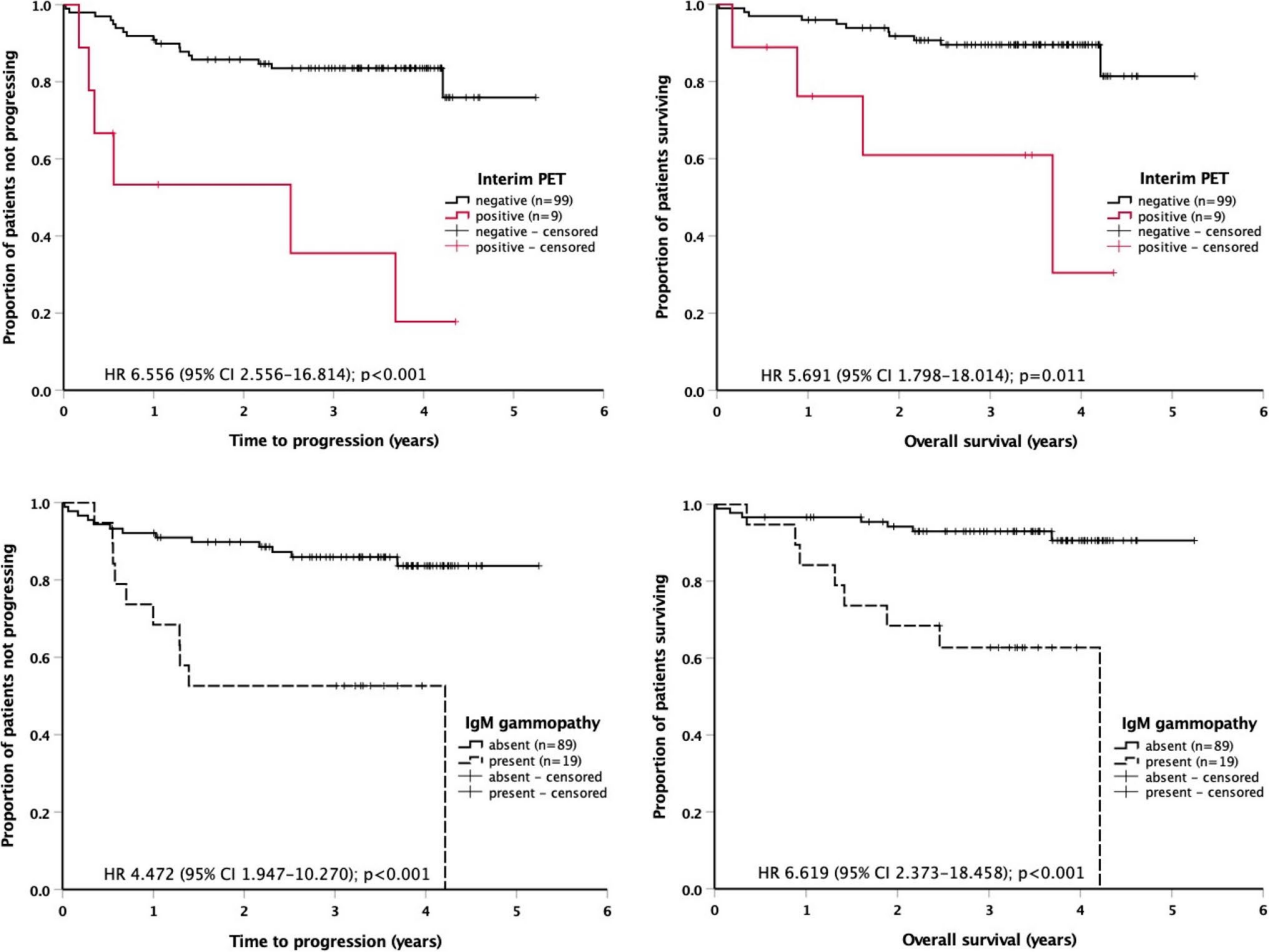
Impact of interim PET response (top) and IgM monoclonal gammopathy (bottom) on time to progression (left) and overall survival (right) in 108 patients with diffuse large B-cell lymphoma. CI, confidence interval; HR, hazard ratio; PET, positron emission tomography

The PET response to the first 2 cycles of R-CHOP did not significantly differ between patients with or without IgM gammopathy (Table 3). The interim PET result was highly predictive of outcome, but, similar to the entire DLBCL population of the PETAL trial [14], the fraction of high-risk patients thus identified was only 8% (Fig. 2, top). At completion of therapy, a significantly larger proportion of patients with than without IgM gammopathy failed to achieve a remission. Long-term outcome of IgM gammopathy–positive patients was also poor, with significantly reduced time to progression and overall survival (Table 3; Fig. 2, bottom). Central nervous relapse (*n*=3) was restricted to patients with IgM gammopathy.

**Fig. 3:**
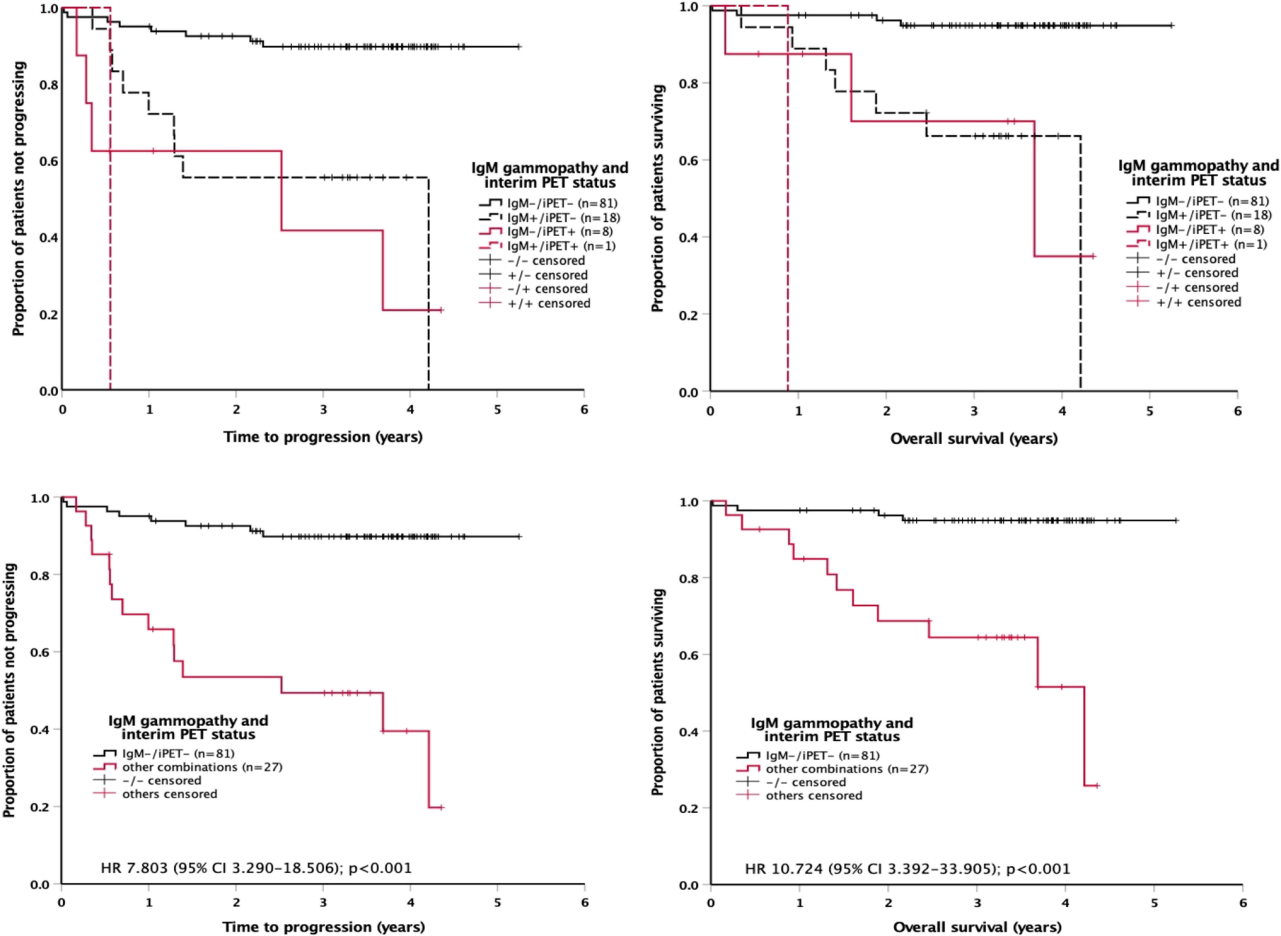
Impact of interim PET response and IgM monoclonal gammopathy on time to progression (left) and overall survival (right) in 108 patients with diffuse large B-cell lymphoma. Top, all 4 possible combinations of favorable and unfavorable results; bottom, two favorable results versus one or two unfavorable results combined. CI, confidence interval; HR, hazard ratio; PET, positron emission tomography

Among IgM gammopathy–positive patients, only one of 19 had a positive interim PET scan. Outcome of interim PET-negative patients with IgM gammopathy was as poor as outcome of interim PET-positive patients without IgM gammopathy (Fig. 3, top). The prognostic information provided by interim PET and IgM gammopathy was subsequently combined (good risk, no risk factor; poor risk, one or two risk factors). The survival curves of the good-risk and poor-risk populations were well separated (Fig. 3, bottom), with a difference in 3-year freedom from progression of 40% (89.8% ± 3.4% versus 49.4% ± 10.0%) and a difference in 3-year overall survival of 30% (94.9% ± 2.5% versus 64.4% ± 9.6%).

### Univariable and multivariable Cox regression analyses

To identify factors impacting outcome systematically, time to progression and overall survival were subjected to univariable Cox regression analysis. Among nine dichotomous variables investigated, only IgM gammopathy, interim PET, and TMTV were statistically significantly associated with outcome (Table 4). The IPI dichotomized into low/low-intermediate risk versus high-intermediate/high risk or low/low-intermediate/high-intermediate risk versus high risk showed no statistically significant correlation. TMTV has previously been reported to outperform the IPI for outcome prediction, suggesting that most of the factors included in the IPI are surrogate markers of tumor burden that can be measured directly by PET [3, 4].

**Table 4:** Univariable Cox regression analysis of the impact of risk factors on time to progression and overall survival

	Hazard ratio	95% confidence interval	*p*
Time to progression (23 events)			
IgM monoclonal gammopathy	4.442	1.947–10.270	<0.001
Interim PET positive	6.556	2.556–16.814	<0.001
Total metabolic tumor volume above prognostic threshold (328 cm^2^)^a^	3.260	1.387–7.665	0.007
IPI high-intermediate or high risk^b^	1.816	0.801–4.116	0.153
IPI high risk^c^	1.605	0.594–4.338	0.351
Age > 60 years	0.947	0.418–2.146	0.895
B symptoms	1.806	0.780–4.182	0.167
Elevated immunoglobulin free light chain concentration	2.126	0.933–4.842	0.072
Monoclonal immunoglobulin free light chain	1.594	0.656–3.877	0.303
Overall survival (15 events)			
IgM monoclonal gammopathy	6.619	2.373–18.458	<0.001
Interim PET positive	5.691	1.798–18.014	0.011
Total metabolic tumor volume above prognostic threshold (328 cm^2^)^a^	4.361	1.478–12.868	0.008
IPI high-intermediate or high risk^b^	1.821	0.660–5.024	0.247
IPI high risk^c^	2.841	0.965–8.361	0.058
Age > 60 years	1.609	0.573–4.523	0.367
B symptoms	2.443	0.883–6.760	0.085
Elevated immunoglobulin free light chain concentration	1.774	0.639–4.928	0.271
Monoclonal immunoglobulin free light chain	1.843	0.630–5.396	0.265

*IPI*, International Prognostic Index; *PET*, positron emission tomography

^a^This threshold was associated with the largest time-to-progression difference in a subset of 510 patients with diffuse large B-cell lymphoma participating in the PETAL trial [3]. Analysis restricted to 94 patients with available data (22 events for time to progression, 15 events for overall survival)

^b^Reference, IPI low or low-intermediate risk

^c^Reference, IPI low, low-intermediate, or high-intermediate risk

Because of low numbers of events, multivariable Cox regression analyses were restricted to three variables at a time [32]. When the three variables showing statistically significant associations with outcome were tested together, the association of IgM gammopathy and interim PET with outcome remained highly significant, while the association of TMTV lost its significance (Table 5). Similarly, when the IPI was tested together with IgM gammopathy and interim PET, only the latter two showed a statistically significant correlation with outcome (Supplementary Information, Tables S3 and S4). The same was true when other variables shown in Table 4 were subjected to multivariable analysis with IgM gammopathy and interim PET as covariates (data not shown).

**Table 5:** Multivariable Cox regression analysis of the impact of risk factors on time to progression and overall survival (subset of 94 patients with available total metabolic tumor volume data)

	Hazard ratio^a^	95% confidence interval	*p*
Time to progression (22 events)			
IgM monoclonal gammopathy	5.443	2.209–13.407	<0.001
Interim PET positive	6.073	2.084–17.698	<0.001
Total metabolic tumor volume above prognostic threshold (328 cm^2^)^b^	1.856	0.730–4.718	0.194
Overall survival (15 events)			
IgM monoclonal gammopathy	8.154	2.643–25.155	<0.001
Interim PET positive	5.478	1.431–20.984	0.013
Total metabolic tumor volume above prognostic threshold (328 cm^2^)^b^	2.520	0.782–8.122	0.122

*PET*, positron emission tomography

^a^Adjusted for the other two covariates

^b^This threshold was associated with the largest time-to-progression difference in a subset of 510 patients with diffuse large B-cell lymphoma participating in the PETAL trial [3]

Elevated free light chains and monoclonal free light chains were associated with reduced time to progression and overall survival, but this association failed to reach statistical significance (Table 4).

## Discussion

Efforts to improve the identification of high-risk patients for risk-adapted therapies have been hampered by the fact that a more precise definition of risk is generally paralleled by a decrease in the number of patients at risk. Models predicting a 50% risk of progression at 3 years often pertain to only 5–15% of patients, with the majority of patients eventually progressing remaining unidentified [2, 6, 33]. In this regard, the combination of interim PET and IgM gammopathy appears to be a good trade-off between risk prediction and population size. The interim PET/IgM gammopathy combination dichotomized the population into a sizeable high-risk group with poor outcome and a three times larger low-risk group with excellent outcome (population size, 25% vs. 75%; 3-year risk of progression, 51% vs. 10%; 3-year overall survival, 64% vs. 95%). This compared favorably with response prediction by interim PET alone (population size, 8% vs. 92%; 3-year risk of progression, 64% vs. 16%; 3-year overall survival, 61% vs. 90%) or IgM gammopathy alone (population size, 16% vs. 84%; 3-year risk of progression, 47% vs. 14%; 3-year overall survival, 63% vs. 93%). It also compares favorably with the 4-tiered IPI (size of high-risk vs. low-risk populations, 10% vs. 52%; 3-year risk of progression or death, 44% vs. 13%; 3-year overall survival, 59% vs. 91%) [2] and the National Comprehensive Cancer Network (NCCN-)IPI (population size, 8% vs. 19%; 5-year risk of progression or death, 70% vs. 9%; 5-year overall survival, 33% vs. 96%) [33].

The major reason for the good performance of the combination is the fact that, except for one patient, the populations identified by a positive interim scan or an IgM gammopathy did not overlap. Despite a negative interim PET scan, the outcome of IgM gammopathy–positive patients was poor. Failure of interim PET to predict outcome has also been reported for lymphomas harboring a *MYC* translocation [34] whose frequency in DLBCL is similar (10–15%) to that of an associated IgM gammopathy [21, 34]. These two abnormalities, however, are unlikely to be related, because, in line with a recent report [10], only one of the IgM gammopathy–positive lymphomas studied here had a *MYC* translocation. In the PETAL trial, MYC translocations were found to be statistically significantly associated with a positive interim PET scan [21].

The frequency of IgM gammopathy among DLBCL patients observed in our study (18%) was in the same range as described previously (13-19%) [7, 8, 10]. Most clinical associations have also been reported before. These include old age, poor performance status, advanced stage, extensive extranodal involvement, elevated lactate dehydrogenase levels, high IPI risk, low remission rates, and poor long-term outcome [7–10]. In addition, we found a highly significant increase in ctDNA and a trend for increased baseline TMTV, markers that are related to IPI and tumor burden [3, 26]. We were unable to reproduce the previously described association of IgM gammopathy with the non-germinal center/activated B-cell-like DLBCL subtype [7, 8, 10] which has been suggested to result from defective class-switch recombination [35]. Neither individual mutations nor the mutation groups defined by the LymphGen classification [27] distinguished IgM gammopathy–positive cases from IgM gammopathy–negative cases.

To identify the origin of the monoclonal protein, we performed IgM and light chain staining in a limited number of available tumor specimens. Immunohistochemistry revealed IgM expression in four, concordant light chain restriction in tumor and monoclonal serum protein in one, and discordant restriction in two of seven samples studied. In two earlier studies, concordant light chain restriction, always associated with IgM expression, was found in 17 of 19 (89%) and 6 of 6 samples (100%; all derived from bone marrow), respectively [8, 9]. In two other studies, immunohistochemistry was restricted to IgM expression that was found in 56 of 63 (89%) and 23 of 41 cases (56%), respectively [7, 10]. Based on our light chain analysis and a previous report [8], at least some patients appear to have an IgM monoclonal protein that is not produced by the tumor. Whether concordant light chain restriction indicates derivation from the lymphoma remains to be demonstrated. Given the clinical associations mentioned above and the lack of distinguishing genetic features, one may speculate that the gammopathy is the product of an immune reaction triggered by a large tumor burden. Similar to gammopathies unrelated to lymphoma [36], the propensity for such a reaction may increase with increasing age.

In contrast to other studies [7, 12, 13], the association of elevated free light chains with poor outcome failed to reach statistical significance. While the frequency of elevated free light chains in our study (36%) was similar to previous reports (19–55%), the overall number of patients analyzed was lower (108 versus 175–409) [7, 12, 13]. Small numbers may explain the observed difference.

The major limitation of our study is its small size. Progression, relapse, or death occurred in a limited number of patients, precluding more extensive multivariable analyses [32]. The observation that interim PET positivity and IgM gammopathy affected largely non-overlapping populations was unexpected and must be confirmed in an independent DLBCL cohort. Strengths of our study include its prospective nature with standardized diagnostic and therapeutic procedures.

In conclusion, interim PET and IgM gammopathy can be combined to dichotomize the DLBCL population into sizeable groups with excellent or poor prognosis. If confirmed in an independent cohort, the interim PET/IgM gammopathy combination may be useful in studies testing risk-adapted treatment strategies.

### Supplementary Information

Below is the link to the electronic supplementary material.

ESM 1 (PDF 295 MB)

## Data Availability

The datasets generated and analyzed during this study are available from the corresponding author on reasonable request.
