# Supplementary material for: Outcome prediction by interim positron emission tomography and IgM monoclonal gammopathy in diffuse large B-cell lymphoma
Source: Ann Hematol. 2023 Aug 11;102(12):3445–55. doi: 10.1007/s00277-023-05393-1 (PMC10640472; doi:10.1007/s00277-023-05393-1)
Supplement: Supplementary file 1 — (PDF 295 MB) [file 277_2023_5393_MOESM1_ESM.pdf]

Supplementary Information

Outcome prediction by interim positron emission tomography and IgM monoclonal gammopathy in diffuse large B-cell lymphoma

Patricia Johansson, Stefan Alig, Julia Richter, Christine Hanoun, Jan Rekowski, Jan Dürig, Bauke Ylstra, Daphne de Jong, Wolfram Klapper, Ash A. Alizadeh, Ulrich Dührsen, Andreas Hüttmann

Table S1.

Pretreatment serum IgM and free light chain concentrations as measured with the IgM Hevylite^{TM} and Freelite^{TM} assays

Table S2.

Gene mutation rates in diffuse large B-cell lymphoma with or without IgM gammopathy

Table S3.

Multivariable Cox regression analysis of the impact of risk factors on time to progression and overall survival (all 108 patients)

Table S4.

Multivariable Cox regression analysis of the impact of risk factors on time to progression and overall survival (all 108 patients)

Table S1.

Pretreatment serum IgM and free light chain concentrations as measured with the IgM Hevylite^{TM} and Freelite^{TM} assays

Pat-ID	HLM kappa	HLM lambda	HLM ratio	HLM-MC	FLC kappa	FLC lambda	FLC ratio	FLC elevated	FLC-MC
1	19.468	0.051	381.725	1	113.625	2.040	55.699	1	1
2	0.886	0.117	7.573	1	13.867	11.633	1.192	0	0
3	4.113	0.652	6.308	1	12.194	6.668	1.829	0	1
4	0.897	0.193	4.648	1	20.537	13.467	1.525	1	0
5	1.010	0.259	3.900	1	11.783	8.978	1.312	0	0
6	0.445	0.132	3.371	1	26.934	14.716	1.830	1	1
7	0.210	0.065	3.231	1	16.718	12.231	1.367	0	0
8	0.314	0.102	3.078	1	18.347	12.153	1.510	0	0
9	0.318	0.106	3.000	1	22.021	13.816	1.594	1	0
10	2.062	0.704	2.929	1	20.190	13.353	1.512	1	0
11	0.291	0.100	2.910	1	44.299	7.424	5.967	1	1
12	0.574	0.488	1.176	1	16.619	13.880	1.197	0	0
13	0.160	0.140	1.143	1	21.881	18.303	1.195	1	0
14	0.141	0.132	1.068	1	22.375	15.843	1.412	1	0
15	0.910	0.982	0.927	1	10.183	8.016	1.270	0	0
16	0.609	0.721	0.845	1	15.230	10.592	1.438	0	0
17	0.281	0.407	0.690	1	54.980	35.686	1.541	1	0
18	1.194	1.921	0.622	1	53.351	38.139	1.399	1	0
19	0.375	1.837	0.204	1	15.327	22.921	0.669	0	0
20	0.559	0.213	2.624	0	16.657	9.225	1.806	0	1
21	0.665	0.255	2.608	0	36.010	21.021	1.713	1	1
22	2.678	1.051	2.548	0	16.403	9.594	1.710	0	1
23	0.289	0.114	2.535	0	25.388	28.011	0.906	1	0
24	0.530	0.221	2.398	0	10.641	8.649	1.230	0	0

25	0.324	0.137	2.365	0	18.969	9.238	2.053	0	1
26	0.701	0.297	2.360	0	15.795	15.072	1.048	0	0
27	0.313	0.134	2.336	0	55.626	22.657	2.455	1	1
28	0.454	0.197	2.305	0	40.720	17.794	2.288	1	1
29	0.242	0.106	2.283	0	26.671	16.108	1.656	1	1
30	0.574	0.252	2.278	0	19.336	12.305	1.571	0	0
31	1.099	0.498	2.207	0	20.693	19.105	1.083	1	0
32	0.238	0.109	2.183	0	25.653	17.372	1.477	0	0
33	0.621	0.292	2.127	0	11.654	9.728	1.198	0	0
34	0.766	0.362	2.116	0	9.099	10.365	0.878	0	0
35	1.813	0.858	2.113	0	13.749	10.503	1.309	0	0
36	0.866	0.425	2.038	0	18.168	18.736	0.970	0	0
37	0.126	0.062	2.032	0	22.791	11.093	2.055	1	1
38	1.488	0.743	2.003	0	22.497	12.522	1.797	1	1
39	0.249	0.125	1.992	0	8.352	7.117	1.174	0	0
40	0.966	0.485	1.992	0	23.070	13.068	1.765	1	1
41	0.910	0.458	1.987	0	25.458	31.870	0.799	1	0
42	0.818	0.413	1.981	0	12.763	9.401	1.358	0	0
43	0.273	0.139	1.964	0	11.501	11.962	0.961	0	0
44	0.768	0.392	1.960	0	11.379	6.554	1.736	0	1
45	0.449	0.230	1.952	0	32.239	15.688	2.055	1	1
46	0.533	0.276	1.931	0	9.292	9.407	0.988	0	0
47	0.128	0.067	1.910	0	12.981	6.384	2.033	0	1
48	0.412	0.218	1.890	0	18.595	15.081	1.233	0	0
49	0.366	0.195	1.877	0	14.613	12.657	1.155	0	0
50	0.473	0.254	1.862	0	18.605	14.678	1.268	0	0
51	0.562	0.304	1.849	0	14.082	10.744	1.311	0	0
52	1.182	0.642	1.841	0	19.310	14.792	1.305	0	0

53	0.651	0.357	1.824	0	18.707	9.792	1.910	0	1
54	0.503	0.276	1.822	0	31.388	19.922	1.576	1	0
55	0.568	0.315	1.803	0	24.411	20.967	1.164	1	0
56	0.462	0.257	1.798	0	13.302	11.736	1.133	0	0
57	0.650	0.362	1.796	0	12.482	12.407	1.006	0	0
58	0.439	0.245	1.792	0	14.136	20.004	0.707	0	0
59	0.077	0.043	1.791	0	8.313	7.405	1.123	0	0
60	0.809	0.452	1.790	0	39.159	22.926	1.708	1	1
61	0.619	0.348	1.779	0	10.851	9.070	1.196	0	0
62	0.686	0.386	1.777	0	10.916	10.670	1.023	0	0
63	0.324	0.183	1.770	0	8.989	11.997	0.749	0	0
64	0.411	0.233	1.764	0	16.250	15.405	1.055	0	0
65	0.423	0.243	1.741	0	11.658	10.926	1.067	0	0
66	0.710	0.408	1.740	0	9.957	7.785	1.279	0	0
67	1.801	1.062	1.696	0	19.468	19.902	0.978	1	0
68	0.465	0.275	1.691	0	22.095	12.758	1.732	1	1
69	0.960	0.581	1.652	0	14.819	13.926	1.064	0	0
70	0.593	0.359	1.652	0	13.214	10.679	1.237	0	0
71	0.334	0.203	1.645	0	23.362	17.294	1.351	1	0
72	0.622	0.380	1.637	0	16.788	12.773	1.314	0	0
73	1.395	0.870	1.603	0	14.428	11.552	1.249	0	0
74	0.318	0.200	1.590	0	16.385	9.940	1.648	0	0
75	0.821	0.520	1.579	0	24.399	15.508	1.573	1	0
76	0.525	0.333	1.577	0	17.830	13.187	1.352	0	0
77	0.115	0.073	1.575	0	7.865	8.164	0.963	0	0
78	0.553	0.353	1.567	0	14.123	10.059	1.404	0	0
79	0.297	0.190	1.563	0	17.362	11.547	1.504	0	0
80	0.504	0.324	1.556	0	22.238	24.093	0.923	1	0

81	0.549	0.354	1.551	0	19.403	9.416	2.061	1	1
82	0.319	0.207	1.541	0	21.049	12.934	1.627	1	0
83	0.836	0.543	1.540	0	12.630	12.882	0.980	0	0
84	0.477	0.310	1.539	0	9.990	8.739	1.143	0	0
85	0.597	0.390	1.531	0	26.784	20.445	1.310	1	0
86	0.740	0.484	1.529	0	9.242	7.072	1.307	0	0
87	0.859	0.568	1.512	0	27.281	27.301	0.999	1	0
88	0.584	0.387	1.509	0	38.508	24.366	1.580	1	0
89	0.096	0.064	1.500	0	16.767	10.123	1.656	0	1
90	0.388	0.260	1.492	0	9.816	6.144	1.598	0	0
91	0.431	0.290	1.486	0	15.418	14.846	1.039	0	0
92	1.213	0.818	1.483	0	29.986	24.308	1.234	1	0
93	0.281	0.192	1.464	0	14.081	9.769	1.441	0	0
94	0.500	0.343	1.458	0	8.053	8.677	0.928	0	0
95	0.137	0.095	1.442	0	8.462	9.331	0.907	0	0
96	0.447	0.311	1.437	0	24.476	24.114	1.015	1	0
97	0.311	0.218	1.427	0	11.524	12.084	0.954	0	0
98	1.732	1.235	1.402	0	22.642	18.427	1.229	0	0
99	0.359	0.262	1.370	0	21.571	12.566	1.717	1	1
100	0.499	0.366	1.363	0	16.626	11.867	1.401	0	0
101	0.476	0.359	1.326	0	18.007	12.561	1.434	0	0
102	0.415	0.315	1.317	0	21.631	15.192	1.424	1	0
103	0.434	0.334	1.299	0	10.214	9.126	1.119	0	0
104	0.111	0.086	1.291	0	8.705	10.295	0.846	0	0
105	0.646	0.514	1.257	0	10.799	11.589	0.932	0	0
106	0.421	0.335	1.257	0	8.251	8.053	1.025	0	0
107	0.796	0.634	1.256	0	24.492	28.788	0.851	1	0
108	0.151	0.123	1.228	0	8.851	13.503	0.655	0	0

Pat-ID, patient identifier; HLM kappa, IgM with kappa light chain (g/L); HLM lambda, IgM with lambda light chain (g/L); HLM ratio, ratio of IgM with kappa versus lambda light chain; HLM-MC, IgM monoclonal gammopathy (ratio outside the reference range [1.18-2.74]), 1 = yes, 0 = no; FLC kappa, free light chain kappa (mg/L); FLC lambda, free light chain lambda (mg/L); FLC ratio, ratio of free light chain kappa versus lambda; FLC elevated, free light chain concentration above reference range (kappa, 3.3-19.4 mg/L; lambda, 5.7-26.3 mg/L); FLC-MC, free light chain monoclonal gammopathy (ratio outside the reference range [0.26-1.65], 1 = yes, 0 = no.

Table S2.

Gene mutation rates in diffuse large B-cell lymphoma with or without IgM gammopathy

	Gene mutation rate				Fisher's
	Absolute		Relative		Exact test
Gene	IgM	No IgM	Ratio IgM	Ratio No IgM	p
TNFRSF14	0/15	10/46	0	0.22	0.055
FOXC1	2/15	1/46	0.13	0.02	0.149
KLF2	2/15	1/46	0.13	0.02	0.149
MYC	2/15	1/46	0.13	0.02	0.149
EBF1	0/15	7/46	0	0.15	0.178
SGK1	0/15	7/46	0	0.15	0.178
CD58	0/15	8/46	0	0.17	0.182
PAX5	4/15	6/46	0.27	0.13	0.243
ARID2	1/15	0/46	0.07	0	0.246
ARID5B	1/15	0/46	0.07	0	0.246
BCL10	1/15	0/46	0.07	0	0.246
HVCN1	1/15	0/46	0.07	0	0.246
MTOR	1/15	0/46	0.07	0	0.246
NUP214	1/15	0/46	0.07	0	0.246
TRAF3	1/15	0/46	0.07	0	0.246
XPO1	1/15	0/46	0.07	0	0.246
ZC3H12A	1/15	0/46	0.07	0	0.246
CDKN2A	2/15	2/46	0.13	0.04	0.251
HIST1H2AM	2/15	2/46	0.13	0.04	0.251
PRDM1	2/15	2/46	0.13	0.04	0.251
NFKBIE	0/15	5/46	0	0.11	0.321
STAT6	0/15	6/46	0	0.13	0.321
CIITA	3/15	4/46	0.2	0.09	0.348
TMSB4X	3/15	4/46	0.2	0.09	0.348
IGLL5	8/15	18/46	0.53	0.39	0.379
ATM	1/15	1/46	0.07	0.02	0.434
FBXO11	1/15	1/46	0.07	0.02	0.434
HIST1H2AC	1/15	1/46	0.07	0.02	0.434
MAP2K1	1/15	1/46	0.07	0.02	0.434
TNFAIP3	1/15	1/46	0.07	0.02	0.434
BCL2	2/15	11/46	0.13	0.24	0.489
EGR2	0/15	4/46	0	0.09	0.564
STAT3	0/15	4/46	0	0.09	0.564
ACTB	0/15	3/46	0	0.07	0.569
DDX3X	0/15	3/46	0	0.07	0.569
EP300	0/15	3/46	0	0.07	0.569
MCL1	0/15	3/46	0	0.07	0.569
MEF2B	0/15	3/46	0	0.07	0.569

NOTCH1	0/15	3/46	0	0.07	0.569
S1PR2	0/15	3/46	0	0.07	0.569
TET2	0/15	3/46	0	0.07	0.569
ARID1A	2/15	3/46	0.13	0.07	0.589
B2M	2/15	3/46	0.13	0.07	0.589
ETS1	2/15	3/46	0.13	0.07	0.589
FOXO1	2/15	3/46	0.13	0.07	0.589
GRHPR	2/15	3/46	0.13	0.07	0.589
ITPKB	2/15	3/46	0.13	0.07	0.589
MPEG1	2/15	3/46	0.13	0.07	0.589
IRF4	2/15	4/46	0.13	0.09	0.630
KLHL14	2/15	4/46	0.13	0.09	0.630
GNA13	1/15	7/46	0.07	0.15	0.666
FAS	1/15	6/46	0.07	0.13	0.670
ZFP36L1	1/15	6/46	0.07	0.13	0.670
BTG2	3/15	6/46	0.2	0.13	0.676
CD79B	3/15	6/46	0.2	0.13	0.676
HIST1H1C	3/15	7/46	0.2	0.15	0.700
SOCS1	3/15	7/46	0.2	0.15	0.700
CREBBP	2/15	9/46	0.13	0.2	0.716
KMT2D	4/15	10/46	0.27	0.22	0.730
PIM1	4/15	15/46	0.27	0.33	0.757
EGR1	1/15	2/46	0.07	0.04	1
HIST1H1B	1/15	2/46	0.07	0.04	1
IL4R	1/15	2/46	0.07	0.04	1
KLHL6	1/15	2/46	0.07	0.04	1
MFHAS1	1/15	2/46	0.07	0.04	1
NFKBIA	1/15	2/46	0.07	0.04	1
OSBPL10	1/15	2/46	0.07	0.04	1
P2RY8	1/15	2/46	0.07	0.04	1
SPEN	1/15	2/46	0.07	0.04	1
TMEM30A	1/15	2/46	0.07	0.04	1
TOX	1/15	2/46	0.07	0.04	1
CCND3	2/15	5/46	0.13	0.11	1
DTX1	2/15	5/46	0.13	0.11	1
TP53	2/15	5/46	0.13	0.11	1
CARD11	3/15	8/46	0.2	0.17	1
MYD88	3/15	8/46	0.2	0.17	1
HIST1H1E	3/15	9/46	0.2	0.2	1
BCL7A	1/15	3/46	0.07	0.07	1
CD70	1/15	3/46	0.07	0.07	1
ETV6	1/15	3/46	0.07	0.07	1
IRF8	1/15	3/46	0.07	0.07	1
MGA	1/15	3/46	0.07	0.07	1

SETD1B	1/15	3/46	0.07	0.07	1
TBL1XR1	1/15	3/46	0.07	0.07	1
VMP1	1/15	3/46	0.07	0.07	1
CD36	0/15	1/46	0	0.02	1
FBXW7	0/15	1/46	0	0.02	1
GNAI2	0/15	1/46	0	0.02	1
IGHG1	0/15	1/46	0	0.02	1
PCBP1	0/15	1/46	0	0.02	1
PLCG2	0/15	1/46	0	0.02	1
PTPN1	0/15	1/46	0	0.02	1
REL	0/15	1/46	0	0.02	1
SF3B1	0/15	1/46	0	0.02	1
SIN3A	0/15	1/46	0	0.02	1
TRAF2	0/15	1/46	0	0.02	1
WHSC1	0/15	1/46	0	0.02	1
CD83	0/15	2/46	0	0.04	1
DNMT3A	0/15	2/46	0	0.04	1
IKZF3	0/15	2/46	0	0.04	1
IRF2BP2	0/15	2/46	0	0.04	1
MS4A1	0/15	2/46	0	0.04	1
PDCD1LG2	0/15	2/46	0	0.04	1
PTPN6	0/15	2/46	0	0.04	1
TCF3	0/15	2/46	0	0.04	1
UBR5	0/15	2/46	0	0.04	1
ZEB2	0/15	2/46	0	0.04	1
EZH2	1/15	4/46	0.07	0.09	1
HIST1H1D	1/15	4/46	0.07	0.09	1
NOTCH2	1/15	4/46	0.07	0.09	1
UBE2A	1/15	5/46	0.07	0.11	1
BTG1	2/15	7/46	0.13	0.15	1
BCL6	2/15	8/46	0.13	0.17	1

No mutations were found in BCL11A, BCL2L1, BIRC3, BRAF, BTK, CCND1, CD19, CD274, CD79A, CDKN2B, CHD2, CSF2RB, CTNNA2, CXCR4, EGR1, HIST1H2BC, HIST1H2BK, HIST1H3B, ID3, IGHG3, IKBKB, ITGB2, KRAS, LTB, PDCD1, PIK3CA, POT1, PTEN, RHOA, RIPK1, RPS15, SAMHD1, SMARCA4, TFAP4, and U2AF1.

Table S3.

Multivariable Cox regression analysis of the impact of risk factors on time to progression and overall survival (all 108 patients)

	**Hazard ratio[a]**	**95% confidence interval**	**p**
**Time to progression** (23 events)			
IgM monoclonal gammopathy	6.469	2.478 – 16.886	<0.001
Interim PET positive	10.114	3.516 – 29.088	<0.001
IPI high-intermediate or high risk[b]	0.714	0.277 – 1.843	0.487
**Overall survival** (15 events)			
IgM monoclonal gammopathy	11.860	3.545 – 39.677	<0.001
Interim PET positive	12.963	3.265 – 51.474	<0.001
IPI high-intermediate or high risk[b]	0.559	0.173 – 1.813	0.333

IPI, International Prognostic Index; PET, positron emission tomography
[a] Adjusted for the other two covariates
[b] Reference, IPI low or low-intermediate risk

Table S4.

Multivariable Cox regression analysis of the impact of risk factors on time to progression and overall survival (all108 patients)

	**Hazard ratio**[a]	**95% confidence interval**	**p**
**Time to progression** (23 events)			
IgM monoclonal gammopathy	5.847	2.336 – 14.632	<0.001
Interim PET positive	9.133	3.326 – 25.081	<0.001
IPI high risk[b]	0.853	0.290 – 2.509	0.772
**Overall survival** (15 events)			
IgM monoclonal gammopathy	8.202	2.599 – 25.885	<0.001
Interim PET positive	8.989	2.512 – 32.169	<0.001
IPI high risk[b]	1.479	0.453 – 4.821	0.517

IPI, International Prognostic Index; PET, positron emission tomography
[a] Adjusted for the other two covariates
[b] Reference, IPI low, low-intermediate, or high-intermediate risk
